# Cross-country differences in household healthcare spending

**DOI:** 10.1093/haschl/qxag234

**Published:** 2026-09-03

**Authors:** Irene Papanicolas, Jonathan Cylus, Maecey Niksch, Luca Lorenzoni

**Affiliations:** Professor of Health Services, Policy and Practice, Brown University School of Public Health, 121 S. Main St., Box G-S121-8, Providence, RI 02912, United States; Department of Health Policy, London School of Economics, London WC2A 2AE, United Kingdom; Professor of Health Services, Policy and Practice, Brown University School of Public Health, 121 S. Main St., Box G-S121-8, Providence, RI 02912, United States; Department of Health Policy, London School of Economics, London WC2A 2AE, United Kingdom; European Observatory for Health Systems and Policies, London WC2A 2AE, United Kingdom; World Health Organization Barcelona Office for Health Systems Financing, Barcelona 08025, Spain; Professor of Health Services, Policy and Practice, Brown University School of Public Health, 121 S. Main St., Box G-S121-8, Providence, RI 02912, United States; Organization of Economic Cooperation and Development, Paris 75016, France

**Keywords:** affordability, financial hardship, out-of-pocket healthcare payments, health financing

## Abstract

**Introduction:**

Health systems face financial pressures as populations age and economic growth slows, with implications for who pays for health care and for what drives expenditure growth.

**Methods:**

We examined changes in outpatient and pharmaceutical care financing across OECD countries from 2000 to 2023.

**Results:**

While overall health spending growth slowed across countries after the Great Recession, household spending grew modestly everywhere, averaging about half a percentage point growth annually. The United States stood out for persistently high out-of-pocket financing burden—over twice that of most peer countries—and for price growth, rather than utilization, driving out-of-pocket spending for pharmaceutical care. Other countries saw out-of-pocket spending driven more by volume growth and slower price growth.

**Conclusion:**

These patterns highlight potential affordability pressures from out-of-pocket healthcare payments, particularly in the US Policymakers should regularly monitor financial hardship from out-of-pocket payments and reconsider using cost-sharing to address health financing sustainability challenges.

## Introduction

Across countries, health expenditures have steadily risen due to aging populations, demand for care, greater societal expectations, and increased availability of new, expensive technologies.^1^ While these trends create pressure to increase health spending, additional economic, fiscal, and in some cases demographic constraints impede publicly financed health systems from raising commensurate funds.^2^ As a result, many countries are seeking alternative financing sources for health care or ways to slow public expenditure growth. One commonly discussed policy option in response to health system financing pressures is to increase patients’ out-of-pocket contributions toward the cost of care, including through greater out-of-pocket spending at the point of use. Although such policies may relieve pressure on public budgets, this can increase unmet need and expose households to financial hardship, particularly if household incomes do not keep pace with rising out-of-pocket requirements.^3,4^

Despite widespread policy concern about rising out-of-pocket healthcare payments for households, systematic comparative evidence on how household out-of-pocket spending has evolved relative to public and compulsory financing and household earnings remains limited. Whether this has accelerated through major economic disruptions, such as the Great Recession and the COVID-19 pandemic, has also not been systematically documented across a broad set of high-income countries over time.

Furthermore, understanding whether households pay a greater portion of healthcare costs out-of-pocket is only part of the policy question; equally important, and comparatively less well-understood, is why household spending on health care is rising. Increases in out-of-pocket spending may reflect higher prices faced by patients for the same quantity of services, greater utilization of services paid entirely or partially out-of-pocket, or some combination of the two. This distinction has direct policy implications: if household spending is rising because of price increases driven by inadequate regulation or expanding cost-sharing requirements, the appropriate policy response differs fundamentally from that if utilization is rising because of increased health needs or expanded treatment options. In practice, policy changes such as limits in public coverage or longer waiting times can affect both price and volume simultaneously and potentially in opposing directions. Distinguishing between these mechanisms is therefore critical for interpreting trends in household financial burden and designing effective policy responses.

This study used OECD national and health accounts data between 2000 and 2023 across the United States and 9 other high-income countries: Austria, Canada, Denmark, Finland, France, Germany, Spain, Sweden, and the United Kingdom. These countries represent a mix of financing systems, including those funded by general taxation, social insurance contributions, and private insurance premiums. To decompose changes in household out-of-pocket spending into price and volume components, we applied an internationally validated methodology using household final consumption expenditure deflators, which produces estimates consistent with national health-specific deflators where both are available.^5^ Specifically, we focused on outpatient and pharmaceutical care, where household cost-sharing arrangements are most prevalent and policy-relevant. We addressed 3 questions: Over the period 2000-2023, how has the health system financing mix changed across high-income countries, and how does this vary across distinct periods including the Great Recession and the COVID-19 pandemic? How has out-of-pocket spending grown relative to wages? And to what extent has growth in household out-of-pocket spending been driven by changes in utilization vs prices?

## Data and methods

### Data

We used publicly available data from the OECD for the United States and our 9 comparator countries of interest, representing a mix of health systems financed predominantly through different funding streams: Austria, Canada, Denmark, Finland, France, Germany, Spain, Sweden, and the United Kingdom. First, we extracted data from the *Health expenditures and financing* dataset on health care financing schemes for the years 2000-2023, measured in national currency per person. To compare growth in household out-of-pocket spending relative to wage growth, we also utilized average annual wages per full-time equivalent employee, measured in national currency units, from the OECD *Average annual wages* dataset.

To examine trends in health prices households face across countries, we used OECD national accounts data on household final consumption expenditure by function—outpatient and pharmaceutical care in particular—and related deflators. These are available from the OECD database (https://stats.oecd.org) and produced by national statistical authorities following the system of national accounts collection framework.

### Methods

First, we calculated average annual expenditure growth rates for all government and compulsory healthcare financing schemes (ie, the sum of transfers from government domestic revenue, social insurance contributions and other compulsory pre-payments) and for household out-of-pocket payments (ie, costs at the point of care, not including premiums). We examined growth rates descriptively for the overall study period (2000-2023) and 4 sub-periods corresponding to distinct macroeconomic periods: 2000-2008 (pre-Great Recession), 2009-2013 (Great Recession), 2014-2019 (pre-COVID-19), and 2020-2023 (COVID-19). Because the periods were defined around these economic disruptions, rather than by a fixed interval length, their durations differ. Some countries had limited data availability: Canada 2006-2023, Denmark and Sweden 2011-2023, and Spain 2003-2023. Additionally, changes in the OECD System of Health Accounts classification of financing schemes create discontinuities in the series for several countries, including Germany in 2009, the Netherlands in 2006, and the United States in 2014. These breaks reflect changes in the classification of financing arrangements with the OECD data. To address this, we took the mean of the average annual growth rates pre-break and post-break.

Next, we calculated average annual growth rates for wages per full-time equivalent employee for each country and period. We examined the percentage-point difference in average annual growth between household out-of-pocket spending per capita and wages per full-time worker.

Lastly, to examine changes in household out-of-pocket spending for outpatient and pharmaceutical care, we used household final consumption expenditure deflators to decompose health spending into price and volume components, building on prior work validating this approach for international comparisons.^5^ We extended this validated methodology to a broader set of 10 countries and applied it to household out-of-pocket spending on outpatient and pharmaceutical care. Specifically, we used implicit price deflators constructed from household final consumption expenditures on health to produce estimates of price and volume growth consistent with national, health-specific deflators. For the 5 countries where national health deflators exist and validation has been demonstrated (Canada, France, the Netherlands, United States, and Australia), results can be interpreted with greater confidence; for the remaining countries, we relied on cross-national consistency of the national accounts framework as the basis for comparability. Average annual growth rates were then calculated for household final consumption expenditures at constant 2020 prices (volume) for outpatient care and for pharmaceuticals in each country and period.

## Results

### Changes in spending by healthcare financing schemes across countries, 2000-2023

**Figure 1.**
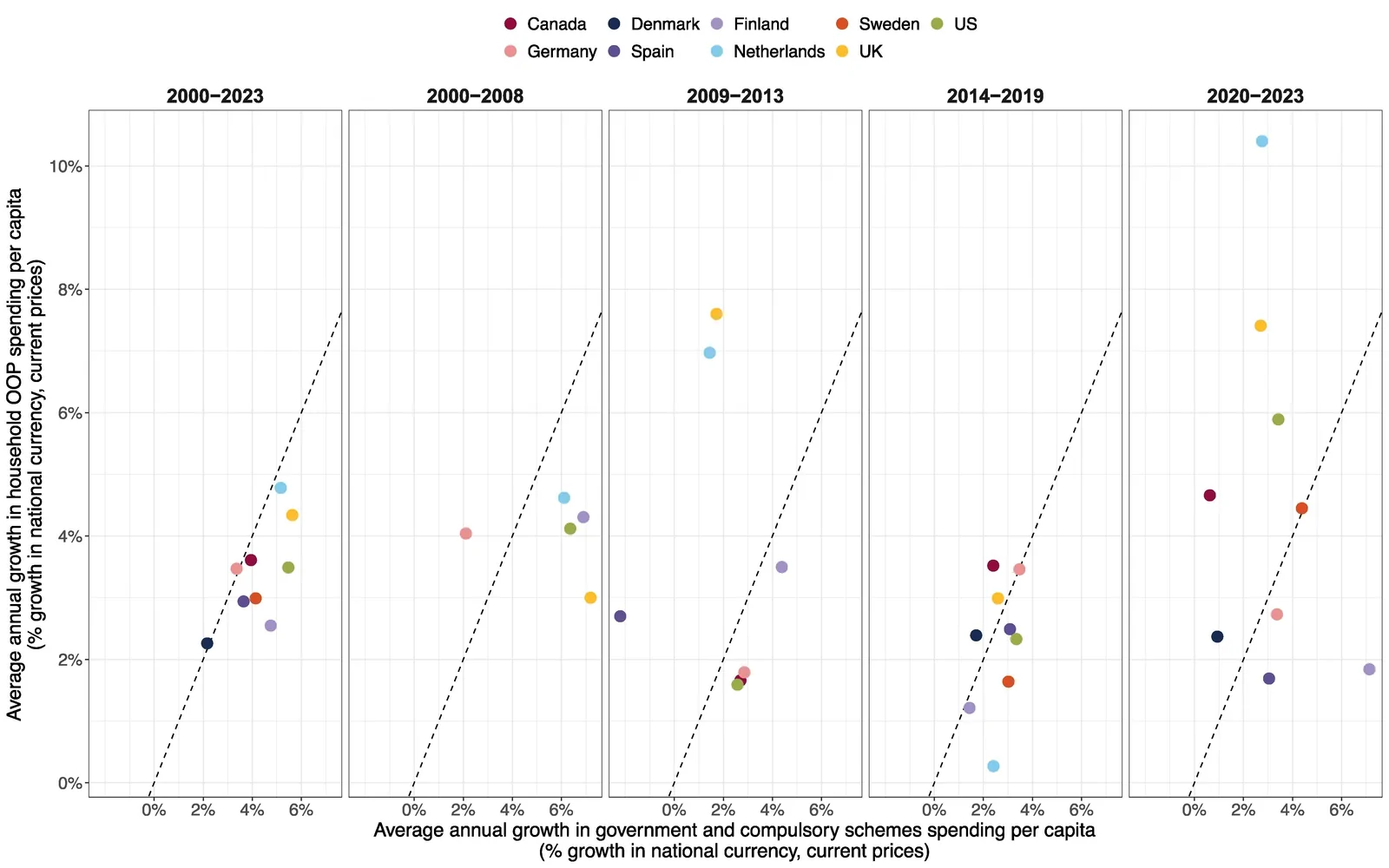
Average annual growth in per capita health revenues by financing source: all government and compulsory schemes vs household out-of-pocket payments, 2000-2023 (% growth in national currency, current prices). Source/notes: Authors’ analysis of OECD data. Each point represents a country. The horizontal axis shows average annual growth in per capita revenues from all government and compulsory contributory schemes (the sum of transfers from domestic government revenues, social insurance contributions, and other compulsory pre-payments); the vertical axis shows average annual growth in per capita out-of-pocket (OOP) spending. The dashed 45-degree line indicates equal growth across financing sources; points above the line indicate faster growth in OOP spending, while points below indicate faster growth in government and compulsory revenues. Austria and France are excluded due to missing revenue data. There are breaks in the series for several countries. Germany transitions from voluntary to compulsory contributory schemes in 2009; the Netherlands in 2006; and the United States in 2014. Any country without data for the full period was excluded from that period: revenue data for Canada began in 2006, so Canada is excluded from the 2000-2008 panel; for Denmark and Sweden, revenue data began in 2011, so Denmark and Sweden are excluded from both the 2000-2008 and 2009-2013 panels; and for Spain, revenue data began in 2003, so Spain is excluded from the 2000-2008 panel. However, these countries are still included in the 2000-2023 panel, wherein the average annual growth across the full period of available data is reported (Canada 2006-2023, Denmark and Sweden 2011-2023, and Spain 2003-2023).

Figure 1 plots average cross-country annual growth in per capita financing sources of all government/compulsory schemes against growth in household out-of-pocket spending. The 45-degree line indicates equal growth across financing sources; points above the line reflect faster growth in out-of-pocket spending, while points below indicate faster growth in government/compulsory revenues.

Over the full study period (2000-2023), most countries experienced similar growth across financing sources or slightly faster growth in government/compulsory spending than in household out-of-pocket spending. This finding was also reflected in 2000-2008 for all countries except Germany.

Different patterns emerged for later sub-periods. During the Great Recession (2009-2013), out-of-pocket growth outpaced that of public and compulsory revenue sources in several countries—including the United Kingdom, the Netherlands, and Denmark. In the post-recession period (2014-2019), growth rates converged across financing sources, with most countries clustering close to parity. The Netherlands was a notable exception, with government and compulsory revenues exceeding nearly null out-of-pocket spending growth. Finally, during the pandemic period (2020-2023), many countries, including the United States, exhibited relatively faster growth in out-of-pocket spending compared to government and compulsory revenues. Appendix Figure A1 presents average levels of household out-of-pocket spending per capita (PPP-adjusted USD) across countries and periods. Notably, the United States consistently had the greatest levels of household out-of-pocket spending across all periods, at more than twice the OECD average.

### Household out-of-pocket spending growth relative to wage growth across countries, 2000-2023

**Figure 2.**
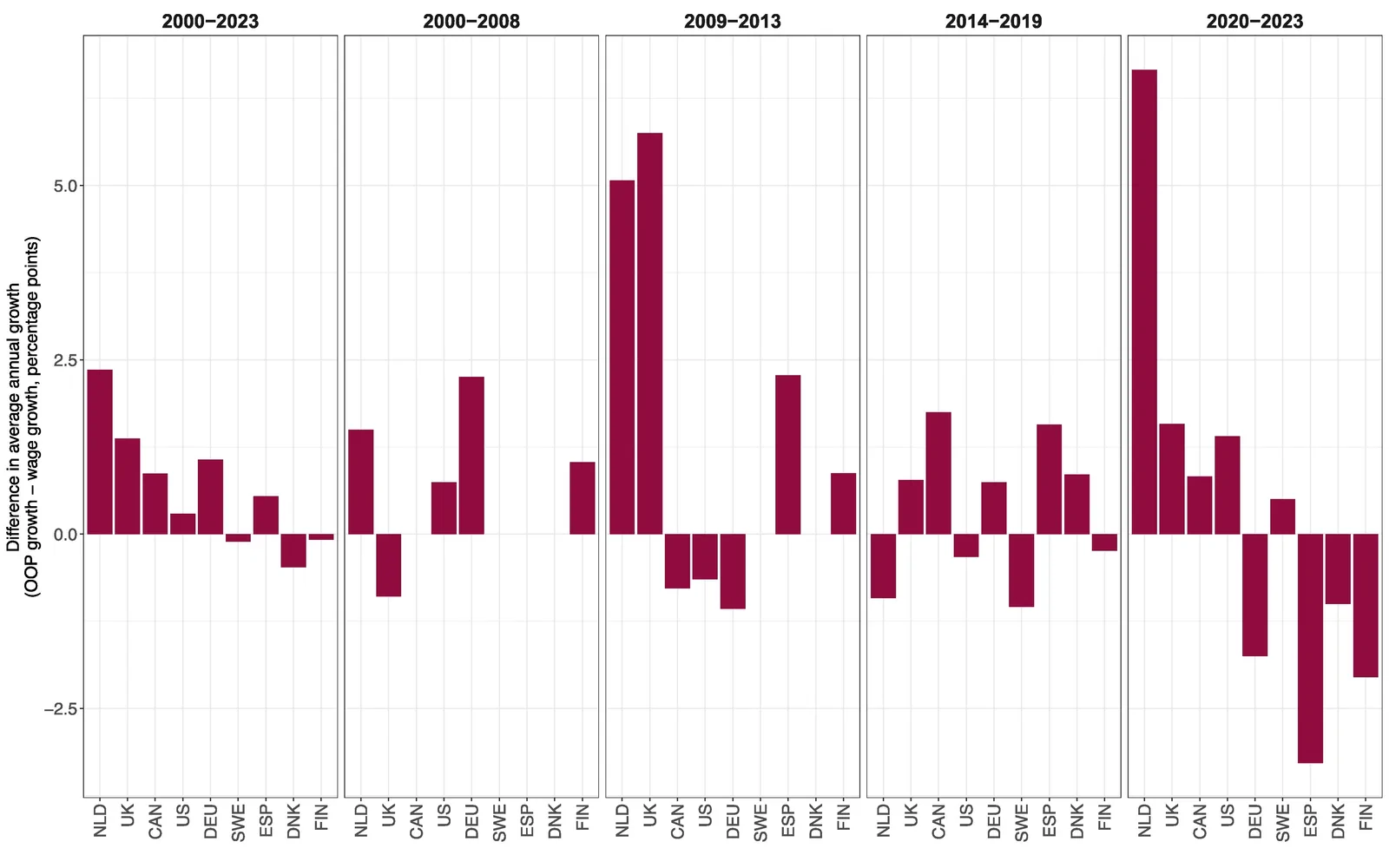
Average annual growth in out-of-pocket (OOP) per capita revenues vs average annual growth in wages per worker (2000-2023) (% growth in national currency, current prices). Source/notes: Authors’ analysis of OECD data.

Next, we compared average annual out-of-pocket spending growth to average annual wage growth across countries and time periods (Figure 2). Across the full study period (2000-2023), out-of-pocket spending increased at a faster percentage rate than wages in most countries. Denmark, Finland, and Sweden were notable exceptions, where wage growth generally kept pace with or exceeded out-of-pocket spending growth.

This relationship varied across sub-periods. In 2000-2008, out-of-pocket spending growth exceeded wage growth in all countries except the United Kingdom. During the Great Recession (2009-2013), this pattern weakened, with several countries—including Canada, Germany, and the United States—experiencing slightly faster wage growth than out-of-pocket growth. In the post-recession period (2014-2019), out-of-pocket spending growth outpaced wage growth in about half of study countries, and wage growth slightly exceeded out-of-pocket spending growth among the others. During the pandemic period (2020-2023), out-of-pocket growth substantially exceeded wage growth in the Netherlands and in the United Kingdom and the United States with smaller magnitudes, whereas other countries exhibited closer alignment or slower out-of-pocket spending growth relative to wage growth. These patterns indicate that the relationship between household out-of-pocket health care payments and earnings is both country-specific and highly sensitive to macroeconomic conditions.

### Contributions of price and volume toward out-of-pocket payments growth faced by households for outpatient and pharmaceutical care, 2000-2023

Decomposing household out-of-pocket spending into price and volume components revealed distinct patterns across service types and countries that would be obscured by examining spending growth alone.

**Figure 3.**
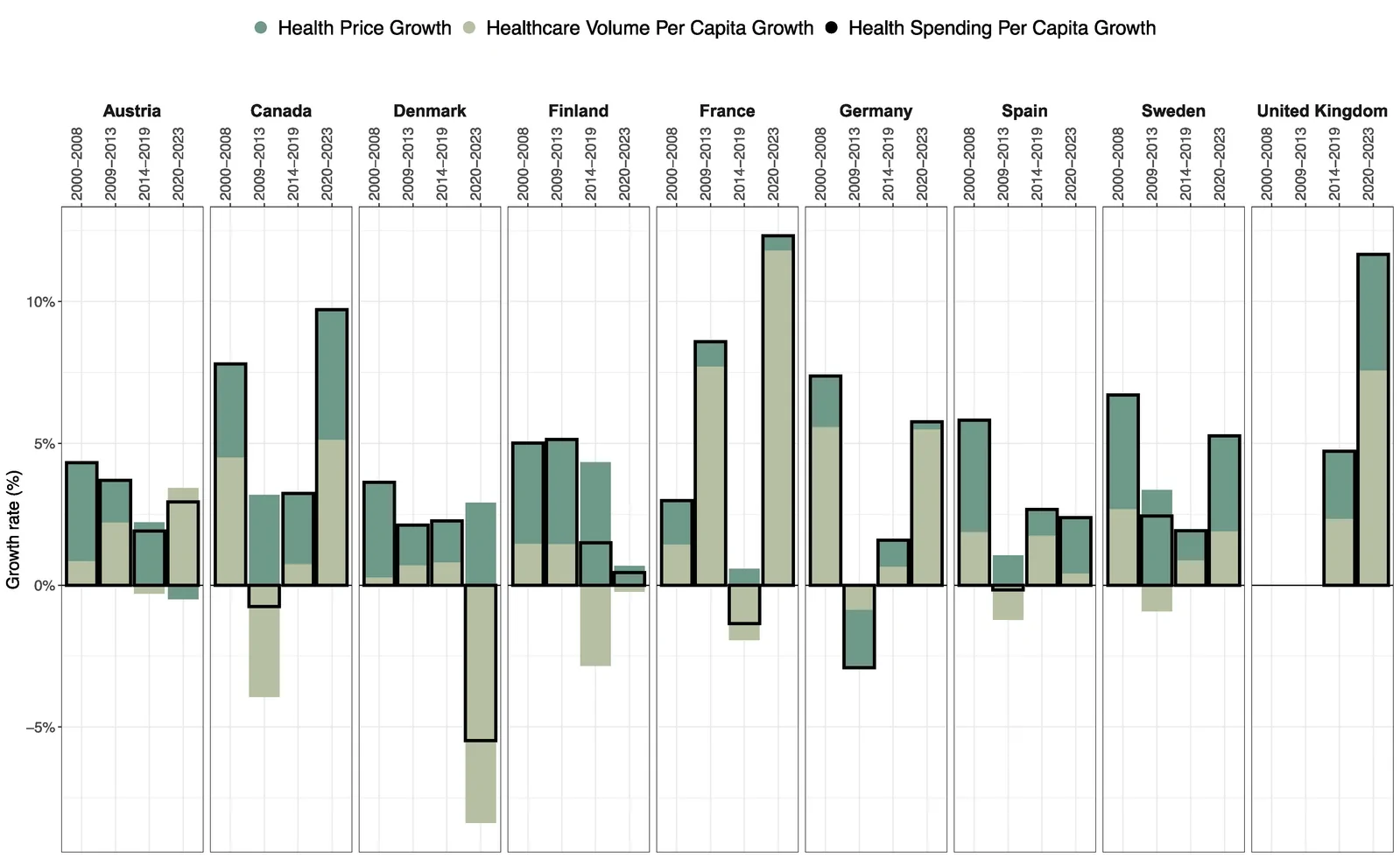
Nominal and real growth in household out-of-pocket (OOP) healthcare spending per capita, outpatient spending (2000-2023). Source/notes: Authors’ analysis of OECD data. Some countries did not have data available for the full time series: Austria 2000-2022, Canada 2003-2023, Denmark 2000-2022, France 2003-2022, Germany 2000-2022, Spain 2003-2022, and the United Kingdom 2013-2023. The United States did not have available data on outpatient expenditures, prices, and volumes. OECD estimates have been externally validated for Australia, Canada, France, the Netherlands, and the United States.

For outpatient care (Figure 3), out-of-pocket expenditure growth was positive in most country-periods and generally reflected contributions from both prices and utilization. However, the relative importance of these components varied substantially across countries and over time. In Austria, Sweden, and Finland, outpatient expenditure growth was primarily driven by price increases in most periods, despite utilization declines. In contrast, Canada, Denmark, France, Germany, Spain, and the United Kingdom generally exhibited growth driven by both prices and utilization. The pandemic period (2020-2023) disrupted these patterns: growth was primarily utilization-driven in Austria, France, and Germany and predominantly price-driven in Sweden, Finland, and Denmark, with Denmark additionally experiencing declining utilization.

**Figure 4.**
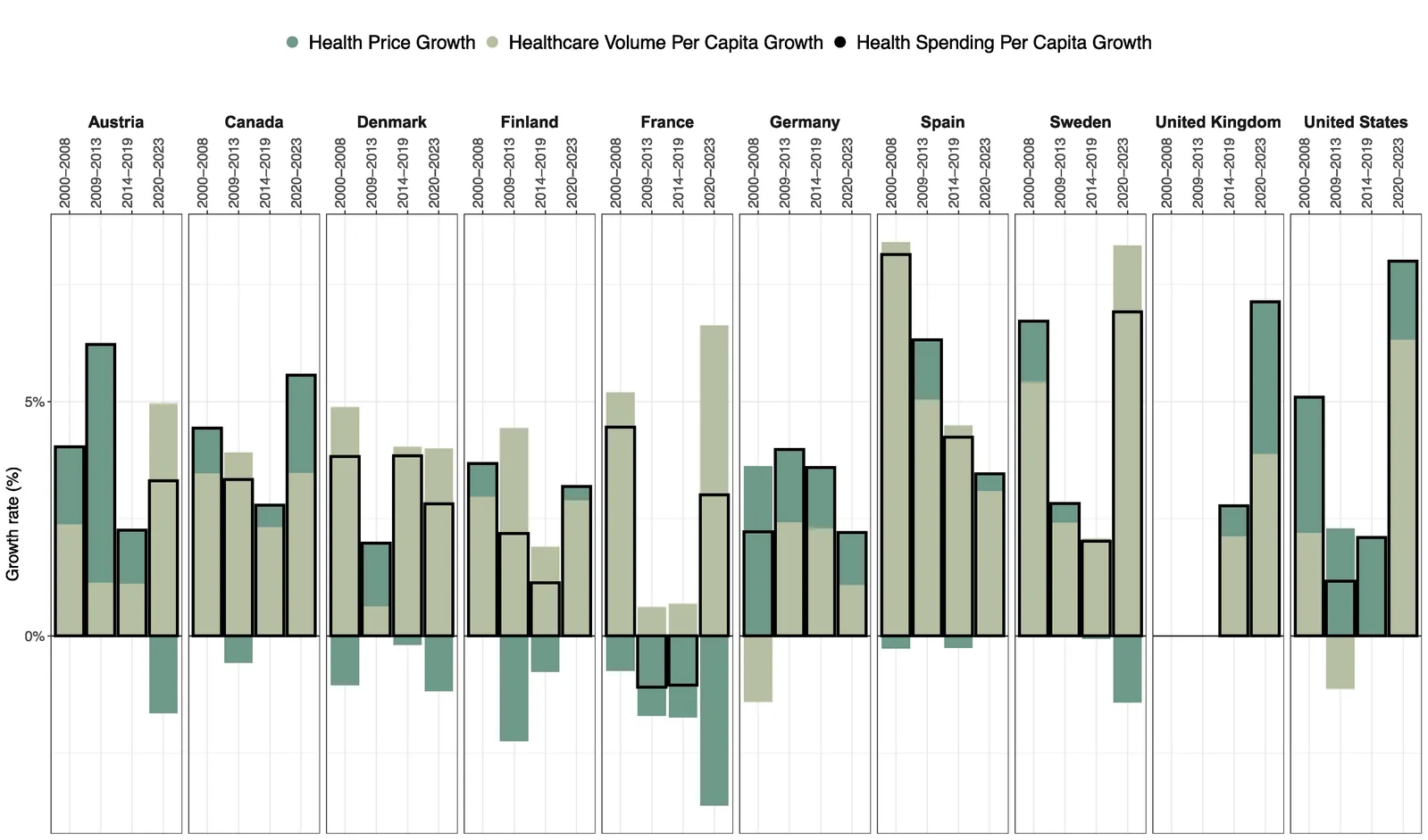
Nominal and real growth in household out-of-pocket (OOP) healthcare spending per capita, pharmaceutical spending (2000-2023). Source/notes: Authors’ analysis of OECD data. Some countries did not have data available for the full time series: Austria 2000-2022, Canada 2003-2023, Denmark 2000-2022, France 2003-2022, Germany 2000-2022, Spain 2003-2022, and the United Kingdom 2013-2023. OECD estimates have been externally validated for Australia, Canada, France, the Netherlands, and the Unites States.

For pharmaceutical care (Figure 4), expenditure growth was generally driven by increases in utilization, rather than prices. In many countries, pharmaceutical prices declined across multiple periods, notably in France. Price growth was most pronounced in Austria, Germany (particularly in 2000-2008), and the United States. In some cases, price increases coincided with declining utilization, including Germany (2000-2008) and the United States (2009-2013 and 2014-2019). In the pandemic period (2020-2023), pharmaceutical care expenditure growth was driven by increases in utilization across all countries. Simultaneously, pharmaceutical prices declined in several countries—including Austria, Denmark, France, and Sweden—while prices increased in the remaining countries.

## Discussion

This study examined the burden of healthcare financing on households across 10 high-income countries between 2000 and 2023. Three key findings emerge from our analysis. First, changes in financing mix indicate that, in many countries and periods, out-of-pocket payments have grown at a similar pace to public and compulsory financing—and often faster than wages. Although these increases are modest in absolute terms, their growth relative to wages implies a potentially meaningful change in the affordability of care over time. Reports of unmet need for medical care across OECD countries substantiate affordability concerns: low-income populations consistently reported greater levels of unmet need than those with higher incomes.^6^

Second, growth in household out-of-pocket spending reflects different underlying dynamics across service types. Outpatient expenditure growth was driven by a combination of price and utilization changes, while pharmaceutical spending growth was primarily driven by utilization increases, often alongside stable or declining prices. These dynamics are likely related to cost-sharing policies, particularly in the outpatient setting.

Third, the United States stands apart not only in the level of out-of-pocket payments but also in the mechanism driving pharmaceutical spending growth: unlike all comparator countries, US household out-of-pocket spending for pharmaceuticals was driven by price increases alongside declining utilization, a pattern that suggests households are paying more while accessing less care.

The patterns described in this analysis provide insight into how healthcare financing pressures are distributed between payers and households. While overall health spending growth has moderated in many countries, out-of-pocket contributions have continued to grow at similar or faster rates than public and compulsory financing in some periods, often outpacing wage growth. Although these increases are modest in absolute terms, their growth relative to wages suggests that out-of-pocket payments at the point of care may be becoming less affordable for households.

Importantly, this does not reflect a general shift in the financing mix toward out-of-pocket payments. Across our study countries, the share of total health spending financed out of pocket declined over the study period, proudly consistent with the wider OECD. Rather, out-of-pocket household payments can continue to increase relative to earnings even as they account for a declining share of health spending. Consistent with increasing health spending relative to household resources, across OECD countries with available data, total health spending increased as a share of household final consumption expenditure from 4.1% of household final consumption expenditure in 2000 to 4.7% in 2010, 5.2% in 2020, and 6.4% in 2024.^7^ Importantly, these comparisons do not capture the full economic incidence of healthcare financing, which households may also bear through taxes, insurance premiums, social contributions, or foregone wages associated with employer-sponsored insurance.

These patterns are particularly evident for outpatient care, where households in many countries experienced price growth above general inflation, especially prior to the Great Recession. The pandemic period (2020-2023) may represent a further inflection point: more countries than in any prior sub-period showed out-of-pocket spending growing faster than public and compulsory revenues. Elevated overall inflation during this period likely further amplified affordability pressures for households from out-of-pocket healthcare payments.

The divergence between price and volume trends provides further insight into the potential mechanisms shaping households’ out-of-pocket healthcare payments. In outpatient care, expenditure growth in many countries was driven in part by price increases, indicating that households were often paying more for care, rather than simply using more services. Furthermore, the relative contributions of price and utilization shifted across periods and countries, often in ways that align with changes in cost-sharing policy.

Three country examples illustrate how different cost-sharing mechanisms shape the price and volume components of household spending in contrasting ways: a case where copayments were introduced, one where they were removed, and one where non-price rationing may increase private spending. In Finland, a sharp decline in utilization growth in 2014-2019 coincided with the introduction and subsequent increase of outpatient user fees in 2016, which were raised by 27.5% to generate additional revenue of 150 million Euros.^8,9^ While we cannot determine if these changes are directly the result of this policy, the timing of the introduction of copayments coincides with the sharp reduction in outpatient utilization observed in our analysis. The utilization reduction of nearly 4 percentage points resulted in minimal overall expenditure growth, despite continued price growth.

Germany's trajectory provides a contrasting example. Until the end of 2012, a “practice fee” (Praxisgebühr) of €10 per quarter existed for any first contact at a physician's office and when other physicians were seen without referral during the same quarter. The fee was introduced to reduce the number of unnecessary physician visits in ambulatory care; however, it proved to be administratively onerous and unpopular among both the general population and medical professionals, leading to its abolition in 2012.^10^ Our analysis shows that during the period when this fee was removed, utilization growth more than offset the decrease in price growth, driving positive overall expenditure growth in household expenditures.

The United Kingdom highlights the role of non-price mechanisms. User fees do not exist for any outpatient visits, as the National Health Service offers free care at the point of service. Long waiting times have coincided with increasing use of privately financed outpatient care, which may contribute to the growth in household expenditure, by both price and utilization, observed in our analysis.^11,12^

These patterns suggest that households likely respond to financial incentives at the point of care and to other forms of more implicit rationing, such as long wait times. The magnitude of these utilization responses also suggests that, despite being an attractive way to raise revenues, copayments may create barriers to care that extend beyond reducing unnecessary services, potentially affecting access to needed care as well. This trade-off should be carefully evaluated by policymakers as they consider introducing user fees.

For pharmaceuticals, we see a very different picture, where expenditure growth was predominantly driven by volume growth. Price growth for households was less pronounced for pharmaceutical care across all periods, likely reflecting policy interventions such as reference pricing, generic substitution programs, health technology assessment, and direct price negotiations that have been implemented across many of these countries to rein in price levels and price growth. In addition, many countries have strict limits on cost-sharing for pharmaceutical care and exceptions for populations in need, which could explain increased utilization without corresponding increases in price growth. The United States is a concerning and notable exception to this: expenditure growth from 2009 onwards reflected only price growth pushed onto households and decreasing utilization. This confirms reports elsewhere of high cost-sharing that often forces households to stop their medications, with important ramifications for their health.^13^

Our findings complement prior research documenting the fiscal sustainability challenges facing health systems, extending this work to examine household-level financial pressures over the past 2 decades. Previous studies have documented differences across countries in spending growth, with comparative analyses frequently focusing on the United States as an outlier.^5,14-16^ Our analysis adds nuance by showing how these pressures manifest differently across service types and demonstrates that the slowdown in public spending growth has not been matched by equivalent moderation in household contributions. This work builds on examinations of cost-sharing by providing comprehensive cross-national evidence on the price and volume components of health spending growth,^5^ suggesting that the mechanisms driving household financial exposure may vary across sectors.

Our analysis has several limitations. First, we focused on aggregated patterns and cannot capture distributional effects across income groups, which have been documented elsewhere.^4,17^ Second, our analysis also focuses on out-of-pocket payments at the point of care and does not include insurance premiums. This distinction is particularly relevant in countries such as the United States and the Netherlands, where households may make substantial premium contributions and where changes in benefit design may alter the balance between premiums and cost-sharing. Consequently, trends in out-of-pocket spending should not be interpreted as capturing changes in households’ total healthcare financing burden. Next, we relied on implicit price deflators, rather than direct price observations, for many healthcare services, which may not fully capture quality changes or shifts in the mix of services delivered. Finally, while we examined multiple time periods to understand how patterns evolved through different economic and policy contexts, the most recent pandemic period represents a unique disruption that may not reflect longer-term and even current trends.

Taken together, these findings have important implications for health policy. The sustained growth in household out-of-pocket payments—particularly through price increases for outpatient care—suggests that greater reliance on patient cost-sharing may erode financial protection without consistently achieving the intended demand-moderating effects of cost-sharing policies. Policymakers should therefore monitor household financial exposure and assess whether current cost-sharing arrangements appropriately balance fiscal sustainability with access to care. At the same time, the relatively stable or declining pharmaceutical prices faced by households indicate that targeted policy interventions can effectively moderate specific components of spending, offering lessons for managing cost growth in other sectors. The United States, which remains an outlier in this domain, highlights the consequences of weaker price regulation and higher cost-sharing for pharmaceuticals.

## Disclaimers

The opinions expressed and arguments employed in the article are solely those of the authors and do not necessarily reflect the official views of the OECD or its member states.

## Supplementary Material

qxag234_Supplementary_Data
