# Supplementary material for: Cross-country differences in household healthcare spending
Source: Health Aff Sch. 2026 Sep 3;4(9):qxag234. doi: 10.1093/haschl/qxag234 (PMC13564218; doi:10.1093/haschl/qxag234)
Supplement: Supplementary file 1 [file qxag234_supplementary_data.zip › Appendix_20AUG2026.docx]

**Appendix to “Cross-Country Differences in Household Healthcare Spending”**

**Figure A1: Average levels by period, household OOP payments per capita, 2000-2023 (PPP-adjusted USD)**

Source/Notes: Authors’ analysis of OECD data.
